# A Comparative Investigation of the Predictive Validity of Four Indirect Measures of Bias and Prejudice

**DOI:** 10.1177/01461672221150229

**Published:** 2023-01-20

**Authors:** Jordan Axt, Nicholas Buttrick, Ruo Ying Feng

**Affiliations:** 1 McGill University, Montreal, Quebec, Canada; 2 Project Implicit, Seattle, Washington, USA; 3 University of Wisconsin-Madison, USA; 4 University of Ottawa, Ontario, Canada

**Keywords:** implicit social cognition, implicit associations, attitudes, stereotypes, predictive validity

## Abstract

Although measures of implicit associations are influential in the prejudice literature, comparative tests of the predictive power of these measures are lacking. A large-scale (*N* > 100,000) analysis of four commonly used measures—the Implicit Association Test (IAT), Single-Category IAT (SC-IAT), evaluative priming task (EPT), and sorting paired features task (SPF)—across 10 intergroup domains and 250 outcomes found clear evidence for the superiority of the SC-IAT in predictive and incremental predictive validity. Follow-up analyses suggested that the SC-IAT benefited from an exclusive focus on associations toward stigmatized group members, as associations toward non-stigmatized group members diluted the predictive strength of relative measures like the IAT, SPF, and EPT. These results highlight how conclusions about predictive validity can vary drastically depending on the measure selected and reveal novel insights about the value of different measures when focusing on predictive than convergent validity.

Implicit social cognition (Greenwald & Banaji, 1995) studies how interpersonal beliefs and behaviors can be shaped by psychological processes that are activated automatically (De Houwer & Moors, 2007). Since the introduction of the concept, a great deal of work has investigated whether such “implicit” processes can be measured consistently. Finding reliable measures of implicit social cognition offers new theoretical insights and aids researchers looking to understand the implications of associations that are under less conscious control; ultimately, the provocative ideas behind theories of implicit social cognition are inert without measures that can assess the construct.

Here, we differentiate between the construct of explicit cognition, which represents more controlled processes that are aligned with conscious goals and typically assessed using *direct* measures like self-report (De Houwer et al., 2009), and implicit cognition, a construct assessed via *indirect* measures that use behavioral approaches to infer the presence of various associations. We also distinguish between a measurement *procedure* (a specific methodological tool), a measurement *outcome* (a participant’s performance on a measure), and a targeted *construct* (the construct meant to be assessed by a measure). Researchers in implicit cognition then use various indirect measures (which have the goal of assessing a larger implicit construct) and analyze the outcomes of these measures (which are believed to be shaped by implicit processes). We also refer to performance on indirect measures as reflecting “associations” based on the structure and logic behind many indirect measures, though we note the consistent evidence that performance on these tasks can be influenced by propositional knowledge (e.g., Cone et al., 2017).

There have been a diverse number of approaches for attempting to measure implicit constructs (Wittenbrink & Schwarz, 2007). The most common approach to measuring implicit cognition comes from using response latencies to infer the relative strength of various associations (Nosek et al., 2011). For example, in the Implicit Association Test (IAT), participants categorize stimuli related to an attribute category (e.g., positive or negative words) as well as stimuli of specific target categories (e.g., images of Black and White people) as quickly as possible using two computer keys. Faster categorization times when, for example, White people and positive words (and Black people and negative words) share a response key compared with when White people and negative words (and Black people and positive words) share a response key is interpreted as reflecting more positive implicit attitudes toward White versus Black people.

Often, indirect measures like the IAT are performance-based and speeded, while direct measures use self-report and are untimed. However, this is more a rule of thumb than a definitional aspect of direct and indirect measurement, as exceptions exist (e.g., asking people to rate how much they like their own name as an indirect measure of self-esteem; Gebauer et al., 2008). Broadly, latency-based indirect measures share a similar approach for assessing the construct of implicit cognition, though there are still important conceptual and methodological differences among them (Bar-Anan & Nosek, 2014). For one, the IAT has produced several variants, such as the Brief IAT (BIAT; Sriram & Greenwald, 2009) or Single-Category IAT (SC-IAT; Karpinski & Steinman, 2006). In an SC-IAT, participants view stimuli from only one target but both attribute categories (e.g., assessing the degree to which Black people are more easily categorized with positive or negative words). Other approaches adopt the logic of semantic priming and infer implicit associations from the speed at which stimuli related to certain attributes (e.g., positive vs. negative words) are categorized following the brief presentation of stimuli from either target category (e.g., Black or White faces). In these evaluative priming tasks (EPT; Fazio et al., 1995), an implicit preference for White versus Black people is inferred when participants are faster at identifying positive words following the presentation of a White (vs. Black) face and faster at identifying negative words following the presentation of a Black (vs. White) face.

The present work investigates a potential consequence of this variability in approaches to measuring the construct of implicit cognition by exploring the degree to which research conclusions are contingent on the indirect measure used. Specifically, we compare how different indirect measures fare in two forms of validity: predictive validity (the degree to which performance on an indirect measure predicts performance on a conceptually-related outcome measure) and incremental predictive validity (the degree to which performance on an indirect measure uniquely predicts performance on a conceptually-related outcome measure, above and beyond performance on a parallel direct measure). We conducted a series of high-powered tests to compare the predictive and incremental predictive validity of four indirect measures across 10 intergroup domains and 250 outcome variables. Measures included the IAT, the SC-IAT, EPT, and the sorting paired features task (SPF), all of which are prominent in the implicit cognition literature (Nosek et al., 2011).

If measures largely reach similar conclusions, it suggests they can be used interchangeably when research questions center on predictive validity. However, if conclusions differ strongly across measures, then the choice of measure may require more justification and forethought. As a result, this work carries both theoretical and practical insights. From a practical perspective, identifying measures that are strong or weak in predictive validity may guide researchers in selecting the measure best suited for their research question. More generally, this work has theoretical implications by aiding our understanding of how indirect measures succeed or fail in assessing implicit cognition.

## Prior Comparisons of Indirect Measures

Several prior investigations have compared multiple indirect measures, though conclusions from this work are limited by the fact that studies generally suffered from low statistical power, a reliance on a flawed analytic strategy when testing for predictive validity (i.e., linear regression), and using only a small number of outcome variables or topics. This previous work has also focused mostly on investigations of convergent validity (i.e., how well indirect measures relate to one another).

The available evidence for how well indirect measures correlate with one another or predict outcomes of interest is somewhat inconsistent. In one study (Bosson et al., 2000), 84 participants completed six measures designed to tap into more automatic components of self-esteem. The indirect measures had a generally weak ability to predict any of the selection criterion variables, such as the degree to which ambiguous statements about oneself were interpreted as positive or negative; and also had poor relations among each other (all *r*’s < .23). This latter finding of poor convergent validity was replicated in a follow-up study comparing five indirect measures of self-esteem (Krause et al., 2011). Similar conclusions were reached in a study investigating nine indirect measures of associations about exercise (Zenko & Ekkekakis, 2019), as analyses found that only one measure was consistently associated with four outcome measures, like exercise frequency. More optimistic findings have come from a comparison of indirect measures of racial associations (Cunningham et al., 2001); here, 93 participants completed two forms of an IAT as well as an EPT, and factor analyses found that each measure loaded onto a shared “implicit prejudice” construct.

However, the most thorough comparison of indirect measures comes from Bar-Anan and Nosek (2014), where over 20,000 participants completed one of seven indirect measures across three topics (self-esteem, race, and politics). Each measure showed some evidence of validity by being reliably associated with other indirect measures of the same topic, though the strength of these associations differed strongly across topics, which were generally low for self-esteem, moderate for race, and high for politics. Across topics, the IAT, SC-IAT, BIAT, and SPF performed similarly in tests of convergent validity. This work also represented the most in-depth comparison of predictive validity to date for multiple indirect measures, but ultimately only contained six outcomes (e.g., experiences of interracial contact), and finding no evidence of variability across measures in predictive validity.

These conclusions were bolstered by a follow-up analysis using the same dataset (Bar-Anan & Vianello, 2018), which used structural equation modeling (SEM) to test how well each measure loaded on to the same “implicit” construct. With the exception of the Affective Misattribution Procedure (AMP; Payne et al., 2005), each of the six indirect measures was more strongly associated with the implicit construct for that topic than an explicit construct made up of several self-report measures. More generally, the conclusion emerging from this work is that several indirect measures show comparable levels of convergent validity in assessing the same construct, and while the capacity to assess this construct varies across topics, there is less variability in measurement quality across measures within any one topic (with the possible exception of evaluative priming).

Although this most recent evidence suggests that many measures perform comparably in tests of convergent validity, these conclusions are hampered by the relative nature of convergent validity tests, as each measure acts as the standard for other measures. As a result, a comparison across several measures with average measurement quality may obstruct the ability to identify particularly strong measures, since tests of convergent validity reward correlations with other measures even if such measures assess content unrelated to the targeted construct. Introducing an external form of validation, such as the capacity to predict conceptually related outcome variables, allows for novel conclusions that are not available when looking strictly at relations among measures.

## Incremental Predictive Validity

One supposed appeal of using indirect measures is their ability to assess mental content distinct from that assessed using self-report (Greenwald & Banaji, 1995), and there is evidence that performance on indirect measures does tap into a distinct, but related, construct than performance on self-report measures (Bar-Anan & Vianello, 2018). A potential benefit of these indirect measures is that they then can be used to predict important outcomes, such as policy support, even after controlling for direct measures like self-reported attitudes or stereotypes.

Claims of incremental predictive validity are common in research using indirect measures. For instance, an SC-IAT measuring evaluative associations toward feminism predicted willingness to join a feminist-related organization, even when controlling for self-reported attitudes toward feminism (Redford et al., 2018). In another example, both a BIAT and AMP assessing racial associations independently predicted an intent to vote for Barack Obama over John McCain (Greenwald et al., 2009).

This evidence of incremental predictive validity has been crucial in the adoption of indirect measures. However, one issue in this work is a reliance on multiple linear regression analyses, where researchers enter both the indirect (e.g., the IAT) and direct measure (e.g., self-report) as simultaneous predictors of an outcome. In large samples that use linear regression, inflated rates of Type I errors can emerge in claims of incremental predictive validity simply as a result of measurement error (Westfall & Yarkoni, 2016). In particular, whenever two measures are (a) correlated with one another, as is the case with direct and indirect measures of attitudes or stereotypes (Bar-Anan & Vianello, 2018) and (b) one of those variables is truly related to an outcome, then it is possible for the unrelated measure to appear as a reliable predictor of an outcome simply because it capitalizes on the error present in the measure that is genuinely predictive of the outcome. The risk of this Type I error only increases in high-powered samples with moderate measurement reliability, conditions that characterize much of implicit social cognition research (e.g., Irving & Smith, 2020; Lundberg & Payne, 2014).

The best method for addressing this issue is through latent variable approaches like SEM, which account for measurement error, and simulation studies have shown strong divergences in conclusions about incremental predictive validity when comparing SEM versus multiple linear regression (Westfall & Yarkoni, 2016), with only SEM being able to properly maintain Type I error rates as sample sizes increase. To date, few studies have used SEM in tests of incremental predictive validity for indirect measures (cf., Axt et al., 2020; Kurdi et al., 2021). The most in-depth examination comes from Buttrick et al. (2020) where an analysis of more than 15,000 participants compared conclusions about incremental predictive validity for SEM versus multiple linear regression across 10 IATs and 250 outcome variables. Results found generally strong agreement in the two analytic approaches, as observed in the high correlation between the standardized coefficients for the IAT in a linear regression analysis and the path coefficient for the implicit construct in SEM (*r* = .98) and that the two approaches reached the same conclusion in 91% of analyses.

Here, we replicate and extend this work by incorporating three additional indirect measures. Expanding this question to other indirect measures has multiple advantages. First, analyzing whether similar rates of agreement between the two analysis strategies emerge among additional measures can illuminate whether conclusions from past work are specific to one measure, the IAT, or apply across multiple measures. Second, this work may identify indirect measures that are particularly well-suited for correlational research as they maximize the chances of finding evidence for predictive validity. Finally, these data have broader implications for theories of implicit cognition, as they may highlight significant variability in performance among measures designed with the shared goal of assessing implicit mental processes.

## Method

### Participants and Procedure

We report all measures and data exclusions. See https://osf.io/y5qut/ for data, materials, analysis syntax, and Online Supplement. Participants were volunteers at the Project Implicit research pool (http://implicit.harvard.edu). The study served as a “background study” that was assigned to participants only after they had completed other available studies for which they were eligible. Participants were randomly assigned to 1 of 10 topics (Age, Arab-Muslim, Disability, Gender-Career, Gender-Science, Race, Religion, Sexuality, Weapons, and Weight), and one of four indirect measures (IAT, EPT, SC-IAT, and SPF). Each study session also included a five-item self-report measure of explicit attitudes or stereotypes, as well as 25 self-report outcome measures. Measures were completed in a randomized order. Demographics were provided when participants registered for the research pool.

Participants were able to complete multiple sessions, and analyses were limited to the first time a participant completed each combination of topic and measure. Data for the primary analysis came from 206,290 study sessions that completed at least one indirect measure, representing 120,882 participants (64.8% female, 70.6% White, 74.8% U.S. citizens, *M*_Age_ = 36.58, *SD* = 15.26). The minimum sample size in our tests of predictive validity was 1,099, which provides more than 95% power to detect an effect as small as *r* = .11, and the median sample size (*N* = 1,308) had 95% power to detect an effect as small as *r* = .10.

### Explicit Attitudes or Stereotypes

Explicit attitudes were measured using the same five-item format as in Buttrick et al. (2020). The first item assessed relative preferences between target group members (e.g., −3 = “I strongly prefer Black people to White people,” +3 = “I strongly prefer White people to Black people”). The following two items were 7-point thermometers assessing liking for each target group (−3 = strongly dislike, +3 = strongly like). The final two items used a slider response to assess positivity toward each target group (−100 = extremely negative, 0 = neutral, 100 = extremely positive). Overall explicit attitudes were scored as the averages of three standardized inputs: the relative preference item, a liking difference score from the second and third items, and a positivity difference score from the fourth and fifth items. Explicit stereotypes had a similar design but instead assessed associations with relevant characteristics (e.g., the degree to which a participant associated males with science and females with arts). See Online Supplement for wording of all items.

### Outcome Variables

Outcome variables were highly similar to those in Buttrick et al. (2020). For each topic, 25 items were divided into five classes: (a) policy support, (b) intergroup motivation, (c) anticipated behavioral or emotional reactions during intergroup interactions, (d) prior

intergroup contact, and (e) group-related beliefs. Seventeen items were replaced from Buttrick et al. (2020) because prior results found they did not reliably correlate with an IAT.^1^

Outcomes were a mix of those used in previous Project Implicit data collections, items taken from pre-existing scales (e.g., Fraboni et al., 1990; Yuker et al., 1970) or items adapted across topics when possible. Any additional items that could not be filled from these sources were created by the researchers. Items therefore varied across topics, with the one exception being motivation, where all items were adapted from the Internal Motivation to Respond Without Prejudice scale (IMS; Plant & Devine, 1998). See Online Supplement for wording of all items.

### Implicit Association Measures

Participants were randomly assigned one of four indirect measures: IAT, SC-IAT, SPF, or EPT. Topics were a mix of attitudes (i.e., associations between target groups and the concepts of “good” and “bad”) or stereotypes (e.g., the Weapons topic examined associations between Black and White people with “weapons” and “harmless objects”). Topics were selected based on prominence in the implicit cognition literature (Greenwald & Lai, 2020).

#### Stimuli

For each indirect measure, the attribute category labels consisted of Good (items: Friend, Smiling, Adore, Joyful, Pleasure, Friendship, Happy, Attractive) and Bad (items: Bothersome, Poison, Pain, Nasty, Dirty, Hatred, Rotten, Horrific), with the exception of the Gender-Career, Gender-Science, and Weapons topic conditions. In these topics, attribute category labels were, respectively, Career (items: Career, Corporation, Salary, Office, Professional, Management, Business) and Family (items: Wedding, Marriage, Parents, Relatives, Family, Home, Children), Science (items: Astronomy, Math, Chemistry, Physics, Biology, Geology, Engineering) and Liberal Arts (items: History, Arts, Humanities, English, Philosophy, Music, Literature), and Weapons (items: images of grenade, axe, cannon, mace, revolver, rifle, sword) and Harmless Objects (items: images of bottle, camera, coke, ice cream, phone, Walkman, wallet). See Appendix for more details about labels and stimuli.

#### Implicit Association Test

We followed the seven-block IAT procedure described by Nosek et al. (2007), which consisted of 120 critical trials. In the IAT, words and/or images appeared one at the time at the center of the screen. Participants were instructed to categorize items into category labels on the top-right and top-left corners of the screen. In Block 1 (a practice block), participants categorized items corresponding to the two groups (e.g., Black vs. White people). In Block 2, participants did the same, but with attribute words (e.g., good vs. bad words). Blocks 3 and 4 combined the first two blocks by grouping, for example, Black faces and good words on one key and White faces and bad words on the other key. Blocks 5, 6, and 7 were the same as Blocks 1, 3, and 4, but the key associated with each group switched sides—for instance, Black faces and bad words were now grouped on one key while White faces and good words were grouped on the other key.

#### Single-Category Implicit Association Test

The SC-IAT consists of one practice block and four test blocks (192 critical trials total, across four blocks), and participants must only categorize stimuli from one target group with the relevant attributes (e.g., good vs. bad words). Categories were selected for the SC-IAT based on prior work arguing that stigmatized groups are more salient than non-stigmatized groups (Langer et al., 1976). Salience was prioritized based off a meta-analysis (Kurdi et al., 2019), which showed greater predictive validity of the IAT for behavior toward stigmatized than non-stigmatized group members. If the IAT is a better predictor of behavior toward stigmatized than non-stigmatized group members, then SC-IATs focused on stigmatized group members may also be more predictive of the outcomes used here.

Specifically, the following groups were selected as the single category in each SC-IAT: *Old People* for the Age topic, *Arab Muslims* for the Arab topic, *Disabled Persons* for the Disability topic, *Female* for the Gender-Science and Gender-Career topics, *Black People* for the Race and Weapons topic, *Judaism* for the Religion topic, *Gay People* for the Sexuality topic, and *Fat People* for the Weight topic. In each 48-trial critical block, 14 trials were related to the target category (e.g., images of older people in the Age SC-IAT), 14 trials were related to the block’s associated attribute (e.g., “good” words in blocks that placed old people and good words with the same response key), and 20 trials were related to the block’s unassociated attribute (e.g., “bad” words in blocks where “good” words and old people shared a response key)

#### Sorting Paired Features Test

The SPF consists of sorting paired stimuli into category pairs that appear in each of the four screen corners. These category pairs include all four possible combinations of attribute and target categories. For instance, in the Race SPF, the four category pairs are Black faces + good words, White faces + good words, Black faces + bad words, and White faces + bad words. The procedure was the same as outlined by Bar-Anan and Nosek (2014), and included 120 critical trials across three blocks.

#### Evaluative Priming Task

The EPT procedure followed the one described by Fazio and colleagues (1995). A total of 180 critical trials were administered within three blocks. An initial block instructed participants to categorize only words (e.g., good and bad words) or stimuli (e.g., photos of weapons or harmless objects). The three critical blocks consisted of categorizing words into two labels, but a task-relevant prime appeared for 200 milliseconds before the target stimuli were shown. For example, in the Age EPT, critical trials consisted of the face from an older or younger adult immediately preceding the good or bad words.

### Data Processing

Indirect measures were processed following Bar-Anan and Nosek’s (2014) recommendations. Measures were scored using a variation of the *D* algorithm, where a participant’s average latency difference score when completing stereotype or prejudice incongruent trials versus stereotype or prejudice congruent trials is divided by the standard deviation of response latencies across all critical trials.

#### Implicit Association Test

For all topics, the IAT was scored based on Greenwald and colleagues’ (2003) *D300* method. We removed trials slower than 10,000 ms and faster than 400 ms and excluded participants with more than 10% of trials faster than 300 ms. There was no built-in penalty for incorrect responses. For attitude topics, higher *D* scores indicate more positive associations toward the category listed in Label 2 versus Label 1 in Appendix. For stereotype topics, higher *D* scores indicate a more stereotype-consistent association (e.g., Male/Career and Female/Family).

#### Single-Category IAT

We used the same response latency exclusion criteria as the IAT and calculated an overall *D* score using the same procedure. For attitude SC-IATs, more positive *D* scores meant more positive evaluations toward the categories listed in Label 1 in Appendix. For stereotype IATs, more positive *D* scores indicated a more stereotype-consistent association (e.g., Female/Arts over Female/Science).

#### Sorting Paired Features Task

Exclusion criteria were identical to those for the IAT. Following Bar-Anan et al. (2009), within each block, we computed a *D* score for each of the four trial types (e.g., *Fat-Good, Fat-Bad, Thin-Good, Thin-Bad*), and then calculated a preference score for each block using the difference between single-category *D* scores. Overall SPF scores were the average of the *D* score difference scores across the three blocks. SPF scores were calculated in the same direction as the IAT.

#### Evaluative Priming Task

EPT sessions with >40% incorrect responses were excluded as well as trials that were two standard deviations away from each participant’s average response latency for the relevant grouping (e.g., within *Fat-Bad* trials). For each block, we computed a single-category *D* score as the difference between the average log-transformed response latencies for each combination of target and attribute (e.g., *Fat-Bad* minus *Fat-Good*) divided by the overall standard deviation among those trials. EPT preference scores were calculated using the difference between these two single-category scores (e.g., the thin *D* score minus the fat *D* score), averaged across the three blocks. EPT scores were calculated in the same direction as the IAT.

## Results

### Data Analysis Strategy

Incremental predictive validity for each indirect measure was calculated using the latent-variable approach of Buttrick et al. (2020). Each indirect measure was broken into four quarters, and structural equation models were built in which each outcome was simultaneously predicted by a latent variable formed by the three explicit attitude measure components and a latent variable formed by the four indirect attitude quarters. Latents were allowed to covary. To test for incremental predictive validity for the indirect measure over and above the explicit construct, these models were compared with models in which the regression pathway from the indirect attitude latent and the outcome measure was fixed to zero; any significant loss of model fit indicates significant incremental predictive validity. We also ran ordinary least squares (OLS) regressions for each outcome, where the outcome was predicted by the direct and indirect measures.

For all relational outcomes (i.e., correlations and regression betas), we present meta-analytic results below. To take the nested nature of the data into account, separate meta-analyses were run for each indirect measure within each topic (i.e., one meta-analysis of responses to the Age IAT, one meta-analysis of responses to the Age SC-IAT); we then meta-analyzed the outcome of these 40 models using Wald-type tests for independent meta-analyses, testing for moderation by the interaction of indirect measure and topic. See https://osf.io/y5qut/ for annotated data analysis scripts.

### Descriptive Statistics and Mean-Level Performance

Table 1 presents means and standard deviations for overall *D* scores among each indirect measure across the 10 topics, as well as alphas for the quartering and one-sample *t*-tests against a neutral value of 0. Generally, measures produced evidence of implicit associations, though effects were on average strongest within the IAT (median *d* = 0.35) compared with the SC-IAT (median *d* = −0.03), EPT (median *d* = 0.07) or SPF (median *d* = 0.25); in fact, for nine of the ten topics, the IAT produced the largest effect size. Notably, for seven topics, the SC-IAT revealed mean-level performance that was negative and therefore counter to what would be considered stereotype or prejudice-consistent associations. That is, SC-IAT performance revealed that participants had more positive than negative associations toward Black people, gay people, fat people, old people, Arab Muslims, and people with a disability, though interpreting a zero value in absolute indirect measures can be more difficult and ambiguous than in relative measures (O’Shea & Wiers, 2020).

**Table 1.** Descriptive and Inferential Statistics for D Scores Within Each Measure and Domain.

Domain	IAT				SC-IAT			
	*N*	Mean (*SD*)	Alpha [95% CI]	*t* Test	*N*	Mean (*SD*)	Alpha [95% CI]	*t* Test
Age	1,341	0.42 (0.37)	0.75 [0.75, 0.76]	*t* = 42.09, *p* <.001	1,379	−0.10 (0.29)	0.68 [0.67, 0.69]	*t* = −12.49, *p* <.001
Arab	1,311	0.04 (0.42)	0.82 [0.82, 0.83]	*t* = 3.29, *p* = .001	1,394	−0.12 (0.29)	0.69 [0.68, 0.70]	*t* = −14.89, *p* <.001
Disability	1,236	0.62 (0.44)	0.85 [0.84, 0.85]	*t* = 49.39, *p* <.001	1,286	−0.07 (0.28)	0.69 [0.68, 0.69]	*t* = −9.05, *p* <.001
Gender-Career	1,389	0.31 (0.36)	0.73 [0.72, 0.73]	*t* = 31.66, *p* <.001	1,360	0.20 (0.25)	0.62 [0.61, 0.63]	*t* = 29.54, *p* <.001
Gender-Science	1,350	0.28 (0.38)	0.76 [0.76, 0.77]	*t* = 26.76, *p* <.001	1,263	0.10 (0.26)	0.67 [0.67, 0.68]	*t* = 13.45, *p* <.001
Race	1,472	0.35 (0.42)	0.81 [0.81, 0.82]	*t* = 31.87, *p* <.001	1,381	−0.14 (0.31)	0.72 [0.71, 0.73]	*t* = −16.79, *p* <.001
Religion	1,196	0.17 (0.45)	0.84 [0.84, 0.84]	*t* = 13.01, *p* <.001	1,353	0.14 (0.29)	0.68 [0.67, 0.69]	*t* = 17.48, *p* <.001
Sexuality	1,335	0.27 (0.44)	0.83 [0.82, 0.83]	*t* = 22.58, *p* <.001	1,410	−0.20 (0.32)	0.76 [0.76, 0.77]	*t* = −24.15, *p* <.001
Weapons	1,445	0.32 (0.40)	0.77 [0.77, 0.78]	*t* = 30.37, *p* <.001	1,408	−0.11 (0.29)	0.70 [0.69, 0.71]	*t* = −14.04, *p* <.001
Weight	1,305	0.48 (0.43)	0.82 [0.82, 0.83]	*t* = 40.61, *p* <.001	1,321	−0.06 (0.28)	0.68 [0.67, 0.69]	*t* = −7.84, *p* <.001
	EPT				SPF			
Age	1,320	0.14 (0.40)	0.42 [0.40, 0.43]	*t* = 12.57, *p* <.001	1,246	0.35 (0.52)	0.56 [0.55, 0.58]	*t* = 24.11, *p* <.001
Arab	1,237	0.01 (0.34)	0.24 [0.22, 0.26]	*t* = 1.53, *p* = .127	1,104	0.04 (0.50)	0.49 [0.48, 0.51]	*t* = 2.38, *p* = .017
Disability	1,226	0.06 (0.34)	0.25 [0.22, 0.27]	*t* = 6.45, *p* <.001	1,099	0.56 (0.55)	0.61 [0.60, 0.62]	*t* = 33.27, *p* <.001
Gender-Career	1,292	0.04 (0.37)	0.34 [0.32, 0.36]	*t* = 3.95, *p* <.001	1,224	0.16 (0.47)	0.42 [0.41, 0.44]	*t* = 12.21, *p* <.001
Gender-Science	1,169	0.05 (0.37)	0.36 [0.34, 0.37]	*t* = 4.76, *p* <.001	1,121	0.14 (0.47)	0.40 [0.39, 0.42]	*t* = 10.09, *p* <.001
Race	1,392	0.24 (0.44)	0.55 [0.54, 0.56]	*t* = 19.95, *p* <.001	1,303	0.27 (0.58)	0.65 [0.64, 0.66]	*t* = 17.07, *p* <.001
Religion	1,288	−0.01 (0.34)	0.24 [0.22, 0.26]	*t* = −0.73, *p* = .467	1,102	0.12 (0.51)	0.51 [0.50, 0.53]	*t* = 7.96, *p* <.001
Sexuality	1,337	0.05 (0.34)	0.28 [0.26, 0.30]	*t* = 5.67, *p* <.001	1,131	0.29 (0.52)	0.57 [0.55, 0.58]	*t* = 18.44, *p* <.001
Weapons	1,326	0.06 (0.35)	0.32 [0.30, 0.34]	*t* = 6.45, *p* <.001	1,402	0.17 (0.49)	0.49 [0.48, 0.51]	*t* = 12.70, *p* <.001
Weight	1,290	0.18 (0.41)	0.48 [0.47, 0.49]	*t* = 15.39, *p* <.001	1,249	0.35 (0.52)	0.54 [0.53, 0.55]	*t* = 23.55, *p* <.001

*Note.* IAT = Implicit Association Test; SC-IAT = Single-Category Implicit Association Test; CI = confidence interval; EPT = Evaluative Priming Task; SPF = Sorting Paired Features.

Table 2 presents the correlation between each indirect measure and the self-report attitude or stereotype measure within each topic. As in prior work (e.g., Bar-Anan & Nosek, 2014), each indirect measure showed meta-analytic evidence of a moderate correlation with parallel self-reported attitudes or stereotypes, though the IAT (meta-analytic *r* = .20 [.18, .22]), SPF (meta-analytic *r* = .16 [.14, .18]) and SC-IAT (meta-analytic *r* = .14 [.12, .17]) showed stronger correlations than EPT (meta-analytic *r* = .083 [.061, .11]), meta-regression main effect of measure QM (*df* = 31) = 1055.60, *p* < .001, for pairwise tests involving the EPT, all *Z*’s > 3.86, *p* < .001 (Tukey-corrected for multiple tests).

**Table 2.** Correlation Coefficient With Self-Report Across All Measures and Domains.

Domain	IAT				SC-IAT			
	*r*	95% CI	*N*	*p*	*r*	95% CI	*N*	*p*
Age	0.13	[0.07, 0.18]	1,312	<.001	0.10	[0.05, 0.16]	1,358	<.001
Arab	0.20	[0.15, 0.25]	1,267	<.001	0.12	[0.07, 0.17]	1,354	<.001
Disability	0.11	[0.06, 0.17]	1,191	<.001	0.07	[0.02, 0.13]	1,252	.011
Gender-Career	0.17	[0.11, 0.22]	1,366	<.001	0.06	[0.00, 0.11]	1,342	.033
Gender-Science	0.20	[0.15, 0.26]	1,300	<.001	0.12	[0.07, 0.18]	1,225	<.001
Race	0.28	[0.23, 0.32]	1,434	<.001	0.20	[0.15, 0.25]	1,345	<.001
Religion	0.15	[0.09, 0.21]	1,147	<.001	0.06	[0.01, 0.12]	1,287	.022
Sexuality	0.35	[0.30, 0.40]	1,296	<.001	0.29	[0.24, 0.34]	1,369	<.001
Weapons	0.22	[0.17, 0.27]	1,395	<.001	0.19	[0.14, 0.24]	1,361	<.001
Weight	0.22	[0.17, 0.27]	1,271	<.001	0.20	[0.15, 0.25]	1,279	<.001
	EPT				SPF			
Age	0.09	[0.03, 0.14]	1,295	.002	0.10	[0.05, 0.16]	1,226	<.001
Arab	0.06	[0.00, 0.12]	1,191	.041	0.16	[0.10, 0.22]	1,071	<.001
Disability	0.06	[0.01, 0.12]	1,173	.029	0.11	[0.05, 0.17]	1,069	<.001
Gender-Career	0.04	[−0.02, 0.09]	1,273	.209	0.09	[0.03, 0.14]	1,202	.002
Gender-Science	0.09	[0.03, 0.14]	1,138	.004	0.09	[0.03, 0.15]	1,093	.002
Race	0.15	[0.10, 0.20]	1,348	<.001	0.31	[0.26, 0.36]	1,264	<.001
Religion	0.06	[0.00, 0.11]	1,236	.043	0.08	[0.02, 0.14]	1,069	.006
Sexuality	0.10	[0.05, 0.16]	1,306	<.001	0.26	[0.20, 0.31]	1,111	<.001
Weapons	0.10	[0.04, 0.15]	1,281	<.001	0.17	[0.12, 0.23]	1,355	<.001
Weight	0.10	[0.04, 0.15]	1,259	<.001	0.19	[0.14, 0.25]	1,217	<.001

*Note.* IAT = Implicit Association Test; SC-IAT = Single-Category Implicit Association Test; CI = confidence interval; EPT = Evaluative Priming Task; SPF = Sorting Paired Features.

### Predictive Validity

**Figure 1.**
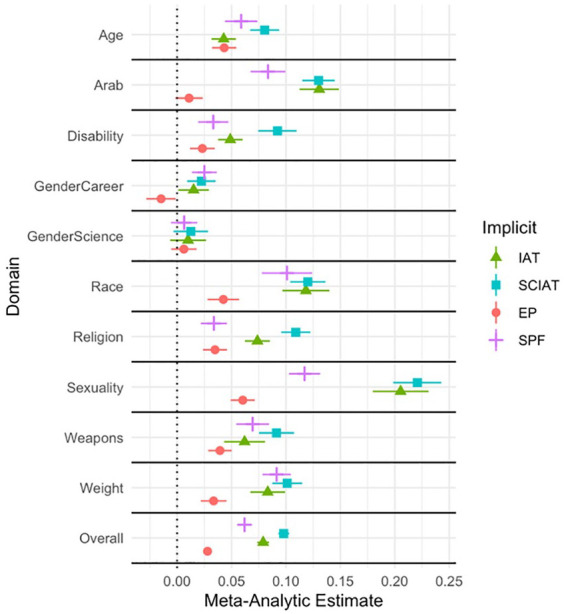
Meta-Analytic Correlations for Each Measure by Outcome Domain. *Note.* IAT = Implicit Association Test; SC-IAT = Single-Category Implicit Association Test; EP = evaluative priming; SPF = Sorting Paired Features.

Table 3 lists the meta-analytic correlation (*r*) between the 250 outcome measures and the parallel indirect measure, as well as information about heterogeneity (see Online Supplement for analyses for each outcome), and Figure 1 presents a forest plot of predictive validity for each measure. Using a cutoff of *p* < .05, the SC-IAT reliably predicted 191 of 250 outcomes (76.4%), which was the highest rate among the four measures (IAT = 59.6%, SPF = 49.6%, EPT = 24.0%). The SC-IAT (meta-analytic *r* = .098 [.093, .10]) had a stronger meta-analytic correlation with the selected outcome variables than the IAT (meta-analytic *r* = .079 [.073, .084]), the SPF (meta-analytic *r* = .062 [.057, .067]), or EPT (meta-analytic *r* = .028 [.024, .032]); meta-regression main effect of measure QM (*df* = 31) = 1,055.60, *p* < .001, all pairwise *Z’s* > 4.66, all *p’s* < .001 (Tukey-adjusted for multiple tests).

**Table 3.** Meta-Analytic Correlation Coefficient for Each Measure in Each Domain Across All Outcome Items.

Domain	IAT		SC-IAT		EPT		SPF	
	*r* [95% CI]	*p*	*r* [95% CI]	*p*	*r* [95% CI]	*p*	*r* [95% CI]	*p*
Age	0.04 [0.03, 0.05]	<.001	0.08 [0.07, 0.09]	<.001	0.04 [0.03, 0.05]	<.001	0.06 [0.04, 0.07]	<.001
Arab	0.13 [0.11, 0.15]	<.001	0.13 [0.12, 0.14]	<.001	0.01 [0.00, 0.02]	.087	0.08 [0.07, 0.10]	<.001
Disability	0.05 [0.04, 0.06]	<.001	0.09 [0.07, 0.11]	<.001	0.02 [0.01, 0.03]	<.001	0.03 [0.02, 0.05]	<.001
Gender-Career	0.02 [0.00, 0.03]	.035	0.02 [0.01, 0.04]	<.001	−0.01 [−0.03, 0.00]	.032	0.03 [0.01, 0.04]	<.001
Gender-Science	0.01 [−0.01, 0.03]	.232	0.01 [0.00, 0.03]	.12	0.01 [−0.01, 0.02]	.295	0.01 [−0.01, 0.02]	.297
Race	0.12 [0.10, 0.14]	<.001	0.12 [0.10, 0.14]	<.001	0.04 [0.03, 0.06]	<.001	0.10 [0.08, 0.12]	<.001
Religion	0.07 [0.06, 0.09]	<.001	0.11 [0.10, 0.12]	<.001	0.03 [0.02, 0.05]	<.001	0.03 [0.02, 0.05]	<.001
Sexuality	0.21 [0.18, 0.23]	<.001	0.22 [0.20, 0.24]	<.001	0.06 [0.05, 0.07]	<.001	0.12 [0.10, 0.13]	<.001
Weapons	0.06 [0.04, 0.08]	<.001	0.09 [0.08, 0.11]	<.001	0.04 [0.03, 0.05]	<.001	0.07 [0.05, 0.08]	<.001
Weight	0.08 [0.07, 0.10]	<.001	0.10 [0.09, 0.11]	<.001	0.03 [0.02, 0.05]	<.001	0.09 [0.08, 0.10]	<.001

*Note.* IAT = Implicit Association Test; SC-IAT = Single-Category Implicit Association Test; CI = confidence interval; EPT = Evaluative Priming Task; SPF = Sorting Paired Features.

### Incremental Predictive Validity

**Figure 2.**
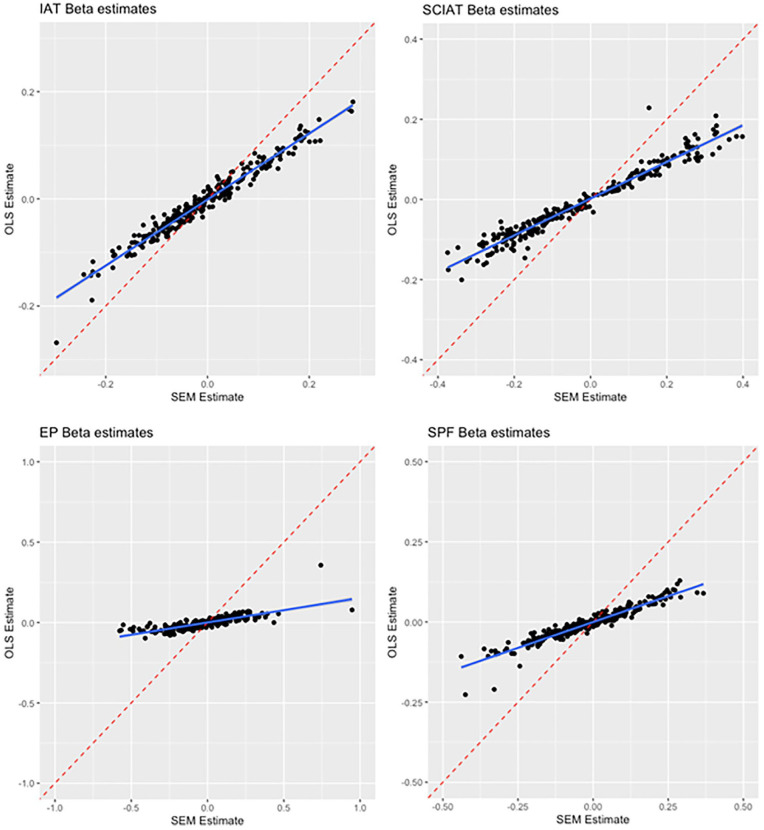
Agreement Between SEM and OLS Incremental Regression Betas, By Measure. *Note.* SEM = structural equation modeling; IAT = Implicit Association Test; SC-IAT = Single-Category Implicit Association Test; EP = evaluative priming; SPF = Sorting Paired Features; OLS = ordinary least squares.

Our first analysis compared the effect sizes and conclusions when between linear regression and SEM analyses of incremental predictive validity. Replicating Buttrick et al. (2020), the two analysis strategies had generally high levels of agreement in terms of rejecting or failing to reject the null hypothesis using a *p* < .05 cutoff. For the IAT, the two analyses reached the same conclusion for 234 of 250 tests (93.6% agreement), and similar rates were observed for the SC-IAT (93.2% agreement), SPF (93.2% agreement), and EPT (92.8% agreement). Figure 2 plots the coefficients that came from the SEM versus linear regression analysis for each of the 250 outcomes across the four indirect measures. The two estimates showed high levels of correspondence for the IAT (*r* = .979 [.973, .984) and a similarly high correlation emerged for the SC-IAT (*r* = .975 [.968, .981]). However, the same was not true for the SPF (*r* = .560 [.469, .640]) or EPT (*r* = −.136 [−.256, −.012). As detailed below, these measures had the lowest levels of incremental predictive validity, which suggests that when most of the estimates are null for a measure, there need not be high correspondence between SEM and linear regression coefficients, since each estimate is being pulled from a null distribution.^2^ Greater agreement between linear regression and SEM coefficients seems to require some level of signal (i.e., predictive validity). See Online Supplement for results of individual analyses.

The second analysis focused on SEM and compared the rate and strength of incremental predictive validity for each measure. Mirroring the results found for predictive validity, the SC-IAT had the highest rate of incremental predictive validity, reliably predicting 159 of 250 outcomes (63.6%). This rate was higher than that observed for the IAT (34.8%), the SPF (22.8%), and the EPT (7.6%), χ^2^(3) = 193.10, *p* < .001. The SC-IAT additionally had the strongest meta-analytic relationship with the outcome measures after controlling for explicit attitudes, SC-IAT meta-analytic *b* = 0.157 [0.147, 0.166], IAT meta-analytic *b* = 0.064 [0.056, 0.073], SPF meta-analytic *b* = 0.098 [0.085, 0.111], EPT meta-analytic *b* = 0.055 [0.032, 0.078], meta-regression main effect of indirect measure QM (*df* = 31) = 531.69, *p* < .001, all pairwise *Z’s* involving the SC-IAT > 7.02, all *p*’s < .001 (Tukey-corrected for multiple tests).

One potential weakness of this analysis is that it is applied to all 250 outcomes rather than those outcomes reliably predicted by the indirect measure. In other words, it may be unreasonable to expect an indirect measure to predict an outcome above and beyond a parallel self-report measure when the indirect measure fails to predict the outcome on its own. To address this point, we ran a follow-up analysis comparing rates of incremental predictive validity limited to outcomes showing a reliable correlation with the indirect measure. Here, the SC-IAT still had the highest rate of incremental predictive validity, reliably predicting 158 of 191 outcomes (82.7%), which was again higher than the IAT (83 of 149 outcomes, 55.6%), the SPF (57 of 124 outcomes, 46.0%), and EPT (16 of 60 outcomes, 26.7%), χ^2^(3) = 80.13, *p* < .001. In addition, when restricting to these outcomes, the meta-analytic strength of relationship between the indirect measures and outcome measures, after controlling for explicit attitudes or stereotypes, was relatively similar across three of four measures: EPT meta-analytic *b* = 0.19 [0.094, 0.29], SC-IAT meta-analytic *b* = 0.18 [0.17, 0.20], SPF meta-analytic *b* = 0.18 [0.16, 0.21], IAT meta-analytic *b* = 0.092 [0.077, 0.11] meta-regression main effect of indirect measure QM (*df* = 30) = 597.59, *p* < .001; with IAT significantly weaker than the other three, all pairwise *Z’s* > 4.05, all *p’s* < .001 (Holm-corrected for multiple tests).

### Agreement Among Measures in Claims of Incremental Predictive Validity

Next, we analyzed the rate at which any two indirect measures agreed with one another in terms of claims of incremental predictive validity, using a *p* < .05 cutoff. For instance, for what percentage of analyses would a researcher have come to the same conclusion concerning the presence or absence of incremental predictive validity, regardless of whether they used an IAT or SC-IAT? Table 4 lists the rate of agreement for every pair of measures, reporting the rate of agreement in rejecting or failing to reject the null hypothesis for claims of incremental predictive validity across all outcomes. In general, rates of agreement were not overly high; for instance, the strongest rate of agreement between any two measures was 74.4%, (EPT and SPF). For the SC-IAT, the highest rate of agreement was 55.2% (with the IAT), and for the IAT, the highest rate of agreement was 64% (with EPT). These results suggest that choice of measure has implications for whether results produce evidence of incremental predictive validity.

**Table 4.** Agreement Rate for Incremental Predictive Validity Between Measure Pairs.

Measure pairing	Conclusion agreement rate
IAT and SC-IAT	55.2%
IAT and EPT	64.0%
IAT and SPF	63.2%
SC-IAT and EPT	37.6%
SC-IAT and SPF	49.6%
EPT and SPF	74.4%

*Note.* IAT = Implicit Association Test; SC-IAT = Single-Category Implicit Association Test; EPT = Evaluative Priming Task; SPF = Sorting Paired Features.

The modest levels of agreement in research conclusions among measures are consistent with two explanations: That each measure does a similarly *poor* job of assessing the targeted construct, or some measures do a superior job than others. To explore this question, we ran an analysis restricted to instances when a measure found evidence of incremental predictive validity. If each measure does a comparably poor job of assessing the implicit construct, there should be similar rates of agreement among measures when restricting to those outcomes that did show evidence of incremental predictive validity, as each measure would be similarly impacted by measurement error. However, if a certain measure provides a *better* assessment of the targeted construct, then that measure should produce high rates of agreement when looking only at cases where incremental predictive validity was found; that is, one measure may largely agree with the other measures any time there is evidence of “signal,” because that measure assesses the construct with less error. Table 5 presents the results of this analysis. The SC-IAT showed superiority; when any of the other three measures found evidence of incremental predictive validity for an outcome, the SC-IAT found incremental predictive validity 76.0% of the time.

**Table 5.** Agreement Rate Between Measure Pairs Limited to Items That Found Evidence of Incremental Predictive Validity.

Outcomes where IAT finds incremental predictive validity	
SC-IAT agreement rate	77.0%
EPT agreement rate	9.2%
SPF agreement rate	29.9%
Outcomes where SC-IAT finds incremental predictive validity	
IAT agreement rate	42.1%
EPT agreement rate	6.9%
SPF agreement rate	28.3%
Outcomes where EPT finds incremental predictive validity	
IAT agreement rate	42.1%
SC-IAT agreement rate	57.9%
SPF agreement rate	31.6%
Outcomes where SPF finds incremental predictive validity	
IAT agreement rate	45.6%
SC-IAT agreement rate	78.9%
EPT agreement rate	10.5%

*Note.* IAT = Implicit Association Test; SC-IAT = Single-Category Implicit Association Test; EPT = Evaluative Priming Task; SPF = Sorting Paired Features.

### Explaining the Superiority of the SC-IAT

#### Psychometric Properties

One explanation for the comparatively high rates of incremental predictive validity in the SC-IAT is that the measure has superior psychometric properties. That is, while each measure assesses the same general construct, the SC-IAT does so with less error, and this reduced error minimizes false positives (Westfall & Yarkoni, 2016). Such an account would not necessarily invalidate any conclusions concerning the superiority of the SC-IAT in tests of incremental predictive validity but would identify why the measure performed so much better than the EPT, IAT, or SPF.

**Figure 3.**
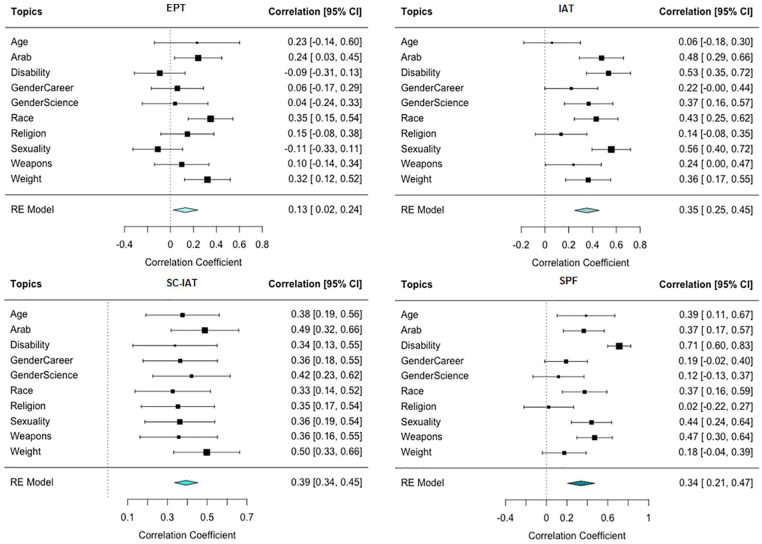
Forest Plots of Test–Retest Reliability for Each Measure. *Note.* CI = confidence interval; RE = random effects; EPT = evaluative priming task; IAT = Implicit Association Test; SC-IAT = Single-Category Implicit Association Test; SPF = Sorting Paired Features.

We investigated this possibility in two ways. First, we analyzed the test–retest reliability of each measure, focusing on the subset of participants who, when completing multiple sessions, were assigned to complete the same topic and measure. In total, 2,878 observations were included in this analysis. Figure 3 presents the forest plots of the test–retest reliability for each measure. Meta-analyzing across topics, EPT showed the lowest test–retest reliability (*r* = .13, 95% CI [.02, .24], *p* =.017), followed by SPF (*r* = .34, 95% CI [.21, .47], *p* <.001), the IAT (*r* = .35, 95% CI [.25, .45], *p* <.001), and the SC-IAT (*r* = .39, 95% CI [.34, .45], *p* <.001). The SC-IAT had stronger evidence of test–retest reliability compared with EPT (*Z* = 4.23, *p* < .001) but not the SPF (*Z* = .74, *p* =.460) or the IAT (*Z* = .14, *p* = .887).

We also investigated construct validity by testing how strongly each measure correlated with other measures in the same topic, focusing on participants who, when completing multiple sessions, completed two measures within the same topic (*N* = 6,834). We meta-analyzed the correlation between each pair of measures, collapsing across topic. These analyses found that the IAT, rather than the SC-IAT, showed the strongest correlations with other measures. For instance, EPT performance was more strongly related to the IAT (*r* = .15, 95% CI [.09, .22], *p* < .001) than the SC-IAT (*r* = .00, 95% CI [−.06, .05], *p* = .913). Similarly, SPF performance was more strongly related to the IAT (*r* = .31, 95% CI [.24, .37], *p* < .001) than the SC-IAT (*r* = .15, 95% CI [.10, .21], *p* < .001). Finally, IAT performance was more strongly associated with the SPF (*r* = .31, 95% CI [.24, .37], *p* < .001) than the SC-IAT (*r* = .26, 95% CI [.21, .32], *p* < .001). In general, analyses of test–retest reliability and convergent validity did not support the account that the SC-IAT has superior psychometric properties.

#### Focus on Stigmatized Group Members

Another explanation for the SC-IAT’s superiority in predictive validity focuses on the measure’s content, as the SC-IAT was the only measure that exclusively focused on stigmatized group members. It is possible that the inclusion of the non-stigmatized or dominant group (White people, straight people, etc.) has limited benefit in terms of enhancing predictive validity; for instance, one prior study (Axt, 2018) found that performance on a racial attitudes IAT was much more related to self-reported warmth toward Black people (|*r*| = .193) than toward White people (|*r*| = .004). Similarly, including majority group members in the indirect measure may assess psychological content that is irrelevant to the content most related to the outcome.

To investigate this idea, we explored whether the rate of incremental predictive validity varied based on whether the outcome did or did not primarily focus on the stigmatized group. We coded each outcome on whether it mentioned only the stigmatized group (e.g., “it is acceptable for companies to have required retirement ages for older employees”), mentioned only the non-stigmatized group (e.g., “younger employees should be given a priority to stay if a company needs to fire some employees”), or mentioned both the stigmatized and non-stigmatized group (e.g., “the driving test given to older people should be more severe than the driving test given to younger people”; see online supplement for coding of each item). A majority of outcomes (77.6%) mentioned only the stigmatized group. Within these items, the SC-IAT (67.7%) showed higher rates of incremental predictive validity than the IAT (35.4%), SPF (22.7%), or EPT (7.5%). In this case, a closer match in content (i.e., only mentioning the stigmatized group) between outcome and measure seemed to have benefited the SC-IAT.

The reverse question can also be asked, concerning whether relative measures (IAT, SPF, EPT) outperformed the SC-IAT among items mentioning both the stigmatized and dominant group members. In a similar test looking at the 28 items that mentioned both groups, the SC-IAT (8/28 or 28.6%) showed comparable or superior levels of incremental predictive validity compared with the IAT (28.6%), SPF (3.6%), or EPT (3.6%).^3^

A final analysis explored whether the superiority of the SC-IAT could emerge from a novel IAT scoring procedure that focused exclusively on trials dealing with stigmatized group members. We re-scored IAT data (the measure with the greatest structural similarity to the SC-IAT), using only trials where stigmatized group members were the focal stimuli (see also Bar-Anan & Nosek, 2014). Despite having one fourth of the critical trials of the SC-IAT, an IAT scoring procedure that only used trials concerning stigmatized group members showed high levels of predictive validity, reliably correlating with 74.8% of outcomes (59.6% when using all IAT trials), and showing incremental predictive validity for 52.0% of outcomes (32.8% when using all IAT trials).

This scoring method showed stronger predictive validity than the scoring method using all IAT data, as multivariate meta-analyses directly comparing the two scoring approaches found that the method using only trials of stigmatized groups correlated more strongly with the outcome measures than when using all IAT trials, meta-regression main effect of analysis approach QM (*df* = 11) = 219.23, *p* < .001; the novel scoring method also had a stronger relationship with outcome measures after controlling for explicit attitudes than did the scoring method using all IAT trials, meta-regression main effect of analysis approach QM (*df* = 11) = 87.47, *p* < .001. Finally, the scoring approach that focused only on stigmatized groups had a stronger incremental relationship with just those outcomes with which they correlated significantly than did the scoring method using all IAT trials, meta-regression main effect of analysis approach QM (*df* = 2) = 89.48, *p* < .001.

However, compared with scores from the traditional SC-IAT, the scoring algorithm that only used IAT trials for stigmatized groups had a weaker overall correlation with the outcome measures, traditional SC-IAT meta-analytic *r* = .098 [.093, .10], stigmatized-only IAT *r* = 0.062 [0.058, 0.065], meta-regression main effect of measure QM (*df* = 11) = 440.83, *p* < .001, had a weaker relationship with the outcome measures after controlling for explicit attitudes, traditional SC-IAT meta-analytic *b* = 0.157 [0.147, 0.166], stigmatized-only IAT *b* = 0.088 [0.078, 0.097], meta-regression main effect of measure QM (*df* = 11) = 267.07, *p* < .001, and had a weaker incremental relationship with just those outcomes with which they correlated significantly, traditional SC-IAT meta-analytic *b* = 0.18 [0.17, 0.20], stigmatized-only IAT *b* = 0.11 [0.097, 0.12], meta-regression main effect of measure QM (*df* = 11) = 365.94, *p* < .001. These results are perhaps unsurprising given how few trials were used in the novel IAT scoring algorithm, but it is still striking that focusing on only a subset of IAT trials could recreate some of the advantages of the SC-IAT.

## General Discussion

In a comparison of four measures of implicit associations (IAT, SC-IAT, EPT, and SPF) covering 250 outcomes and 10 domains, we found that measures of implicit associations should not be treated as interchangeable: Conclusions about incremental predictive validity varied drastically depending on the measure used. Overall, we found that the SC-IAT had the highest rate of both predictive and incremental predictive validity, producing evidence of such validity at nearly twice the rate as the next best measure. In addition, there was only moderate consensus across measures in research conclusions, as no two measures ever reached the same conclusion in more than 75% of analyses.

This research also extends prior work on the incremental predictive validity of indirect measures by using SEM for primary analyses. Whereas most prior investigations of the issue relied on simultaneous linear regression, this approach can potentially inflate Type I errors (Westfall & Yarkoni, 2016). Higher Type I error rates are particularly likely in contexts where the measures being used as predictors have moderate internal reliability and sample sizes are large, which characterizes many prior studies using such measures (Bar-Anan & Nosek, 2014), and a great deal of research in implicit social cognition (e.g., Redford et al., 2018). As a result, we consider these data to be the most accurate and expansive investigation yet into the question of incremental predictive validity for these measures, though below we note several limitations to this work that constrain some of our conclusions.

Results found that one measure—the SC-IAT—had the strongest evidence for predictive and incremental predictive validity. The superiority of the SC-IAT could not be attributed to psychometrics. Instead, the superiority of the SC-IAT in predictive validity seemed most likely due to its exclusive focus on associations toward stigmatized groups. The SC-IAT was more predictive of items mentioning only stigmatized group members, but at the same time the SC-IAT was just as strong a predictor of outcomes that mentioned both stigmatized and non-stigmatized groups. These data suggest that measuring associations toward non-stigmatized groups can lower the predictive power of a measure for outcomes focusing only on stigmatized groups, but also fails to suggest that assessing associations toward non-stigmatized groups can *strengthen* the predictive validity of a measure when the outcome mentions both groups.

The importance of focusing only on associations toward stigmatized groups was seen most clearly when IAT data were reanalyzed to focus only on the 25% of critical trials that presented stimuli related to the stigmatized group. This new analysis of a subset of IAT trials outperformed full IAT data in our tests of predictive and incremental predictive validity (though still performed worse than the actual SC-IAT). Although these data are consistent with a prior study that used a smaller number of outcomes (Bar-Anan & Nosek, 2014), the results are somewhat surprising given that participants completing an IAT are likely in a different state of mind than those completing an SC-IAT, since the IAT requires participants to be aware of stimuli related to non-stigmatized group members. That higher rates of predictive validity could be salvaged from so few IAT trials is a promising avenue for future uses of the measure, which may be able to retain the benefits of measuring associations toward both groups (e.g., when trying to establish a mean-level effect showing more positive associations toward one group than another) but focus on a subset of trials for correlational analyses.

One potential criticism of our conclusions regarding the superiority of the SC-IAT in predictive validity is that the finding arose simply from the fact that most of our outcome measures only mentioned the stigmatized group, and if more of our outcome measures focused on non-stigmatized groups or both groups, relative measures like the IAT could have shown stronger performance. Although this may be true, we argue that much of psychological research on prejudice is itself focused on attitudes or beliefs about stigmatized groups. For example, in a recent analysis looking at the psychometric properties of 25 popular race-related scales (Hester et al., 2022), among items mentioning Black and/or White people, 68.3% of items mentioned only Black people, 22.6% of items mentioned both Black and White people, and only 9.1% mentioned just White people. Similarly, across popular measures of self-reported prejudice concerning religion, age, or disability status (Lee et al., 2009; Rupp et al., 2005; Yuker et al., 1970), 81.5% of items only mentioned stigmatized group members. To the extent that prejudice research is focused primarily on stigmatized group members, the SC-IAT should remain the preferred measure in tests of predictive validity, though whether results extend to other domains (e.g., clinical or consumer settings) is a worthy topic of future research.

A related point is that two topics—attitudes concerning Arab-Muslims and Judaism—had category labels for dominant group members that were of a different of level specificity than for stigmatized group members (i.e., Arab-Muslims vs. “Other People” and Judaism vs. “Other Religions”). These broader category labels may have created difficulties in measurement for the three relative measures. However, removing these two topics from analyses did not alter any conclusions presented here (see Online Supplement for full reporting).

It is sensible that the SC-IAT outperformed the other measures in predictive validity for items only mentioning stigmatized group members, as the relative measures assessed associations toward non-stigmatized group members that appeared to be a diluting force. However, it is less clear why the SC-IAT still performed as well as the IAT or SPF in items mentioning *both* stigmatized and non-stigmatized groups. One possible explanation comes from the linguistic concept of “markedness” (e.g., Irmen & Roßberg, 2004), where some categories are the default and other categories are noted by their deviation from the default (e.g., “male” is unmarked but the “fe” in “female” denotes a marked category). In the outcomes used here, non-stigmatized group members may represent a cultural default that receives little elaboration compared with stigmatized group members, which are a more salient and “marked” category. As a result, even items mentioning both groups may still be more related to associations concerning stigmatized group members, meaning the SC-IAT can remain just as strong a predictor of these outcomes as relative measures. Another possibility is that SC-IATs, by focusing only on a single target category, may minimize the role of polarity effects on performance (Proctor & Cho, 2006). That is, scores on the other indirect tasks may have less validity, as the tasks presented two target categories alongside two opposing attribute categories of positive and negative, which could guide participants to impose a similar contrast between target categories, even if such a contrast was not present in their actual implicit attitudes. These speculative explanations will benefit from more focused studies that could support or refute the framework.

That our measures of behavior were most related to an SC-IAT concerning stigmatized group members potentially stands in contrast to prior work on intergroup discrimination (e.g., Brewer, 1999), which argues that such behavior is driven more by ingroup favoritism than outgroup derogation. Although we lack a definitive explanation for why our measure focused only on stigmatized group members outperformed measures using both dominant and stigmatized members in predictive validity, we note that only a subset of prior studies looking at ingroup favoritism used measures of implicit attitudes and had behavioral outcomes that were able to clearly tease apart ingroup favoritism from outgroup derogation (Greenwald & Pettigrew, 2014). Some related findings to our own come from Kurdi et al (2019), where a meta-analysis found that IATs were slightly more predictive of behavior toward stigmatized than non-stigmatized group members. Resolving this discrepancy between work in implicit social cognition and intergroup processes should be a priority of future studies.

### Implications for Implicit Social Cognition

Though results are specific to the social groups and measures used in the present work, findings have implications for the study of implicit social cognition. First, these data update prior studies of construct validity that investigated relationships among indirect measures (e.g., Zenko & Ekkekakis, 2019), with our results finding generally low correlations between measures, particularly for analyses involving the EPT. Even if these measures may all tap into a general implicit construct (Bar-Anan & Vianello, 2018), our psychometric analyses suggest that it is a measurement approach with a considerable amount of noise (Connor & Evers, 2020).

The present work also carries theoretical implications. These data extend prior investigations of convergent validity in indirect measures (Bar-Anan & Nosek, 2014), which generally found little variation in the degree to which any measure maximized correlations with other measures. Similar results emerged in the present data, specifically for the IAT, SC-IAT, and SPF. However, when using a different criterion of validity, the SC-IAT outperformed other measures in predicting relevant outcomes. This may seem like a paradox: How can three measures correlate similarly with one another, but one correlates better with our outcome measures? One potential answer is that the SC-IAT’s correlation with the other indirect measures may be dampened by its omission of measuring associations toward non-stigmatized groups, but at the same time, this design allows a greater focus on associations toward stigmatized groups, which are intrinsically more related to the outcomes used here. These results highlight novel insights that can be generated from looking beyond convergent validity.

From a practical perspective, this work can help reveal additional considerations for researchers when designing studies and selecting measures. For instance, prior research has stressed the correspondence between indirect and outcome measures. In one example (Irving & Smith, 2020), self-reported preferences for specific fruits (e.g., bananas and cantaloupes) were better predicted by more local IATs (e.g., a cantaloupe IAT) than a global IAT measuring general fruit preferences. Although this past work revealed the importance of a measure’s content, the present study extends this reasoning by illustrating the need to also think critically about what *measure* is most appropriate. In particular, when a research question is focused specifically on predicting attitudes or beliefs about stigmatized group members, there seems to be little benefit in indirect measures that also assess associations concerning non-stigmatized groups.

A similar point comes from the findings of a recent meta-analysis on the predictive power of the IAT, where the IAT was more predictive of relative than absolute outcomes (Kurdi et al., 2019). Given this result, the authors recommend that for researchers interested in using the IAT, they should select outcomes that are also relative. The present work argues for the reverse; if researchers are most interested in an *outcome* that focuses on a single group, they should choose a *measure* that is also centered on associations toward that group. This may be a preferable option because some outcomes are inherently more about the stigmatized group (e.g., certain policy preferences), and in these cases, it will make sense to prioritize the outcome of interest and choose the indirect measure that is most suitable. The present work then echoes prior arguments concerning the overreliance on relative measurement in implicit cognition (O’Shea & Wiers, 2020), and indicates the potential for a greater use of measures or analyses that isolate associations toward one group.

### Is the SC-IAT the “Best” Measure?

Although the SC-IAT showed the strongest evidence of predictive and incremental predictive validity in the topics and outcomes used here, we caution readers against concluding that the SC-IAT is the superior indirect measure in general. This work focused on the relatively narrow question of predictive validity, and the most appropriate indirect measure will always be determined by one’s research question and goals. Indeed, some measures may have strengths that could even limit evidence of predictive validity. For example, past work on the “reliability paradox” (Hedge et al., 2018) finds that measures that maximize an overall, mean-level effect across an entire sample can flatten between-subject variability, thereby suppressing associations with outcome measures. Table 1 shows that the IAT created the strongest mean-level effect for seven of ten topics. As a result, researchers most invested in establishing the presence of an effect (particularly one that could indicate a relative difference in associations between two target categories) would be well-served to prioritize the IAT over the SC-IAT. Subsequent research may find separate strengths for other indirect measures (e.g., those that are most versus least responsive to experimental manipulations).

### Limitations

One limitation of this work is that it only used measures dealing with prejudice and stereotyping, and it is unclear the extent to which results generalize to other domains. Another limitation is that we only included four measures of implicit associations, and future work will be needed to explore the predictive validity of other measures, such as the Brief IAT (Sriram & Greenwald, 2009) or Go No-Go Association Task (Nosek & Banaji, 2001). In addition, while our study was highly powered, it relied on a convenience sample that likely already had a familiarity with the notion of “implicit bias.”

Our conclusions are also limited to the selected outcomes, which cannot be considered a representative sample of outcomes in the prejudice literature. In particular, we relied on self-reported behaviors like intergroup beliefs. This decision was made to maximize sample sizes and the number of predictive validity tests that could be conducted, but it still constrains possible conclusions. Specifically, focusing on self-reported behaviors could limit evidence of predictive validity for indirect measures for outcomes that rely on more automatic processes (such as nonverbal behavior). Future work in this area will surely benefit from efforts to test this question across a wider range of behaviors, even if that means using a smaller number of topics or outcomes, as collecting such behavioral data can be resource intensive.

Primary analyses also collapsed across the intergroup domain, which may have obscured certain domains where measures were particularly likely to be predictive. This analysis decision assumed that each measure functioned similarly across categories, a choice that may not be warranted (e.g., it is possible that the IAT has high validity in the context of race, but low validity for age). It is noteworthy that the SC-IAT did not always show the highest rates of predictive validity across topics, a finding that could either be attributed to the topic (i.e., some topics elicit weak evidence of predictive validity, regardless of measure) or the measure (i.e., an indirect measure can show better or worse capacity to assess the implicit construct across topics). A more thorough understanding of and explanation for variability across topics will benefit researchers in this area, and future studies could make progress on this question by seeing if multiple versions of the same measure create substantive differences in predictive validity within a topic (e.g., whether differences emerge in predictive validity across several different SC-IATs meant to assess age-related implicit attitudes).

We also collapsed across outcome category, which could have obscured variation in predictive validity across outcomes (e.g., beliefs vs. policy support). To investigate this issue, we fit a logistic model predicting whether or not each analysis demonstrated incremental predictive validity by outcome class, topic, and measure. Results, which are reported in the Online Supplement, found evidence of moderation by both domain and by outcome class (e.g., whether the outcome was a belief, about contact, etc.). This moderation will be of interest to future research looking to identify domains or outcomes where indirect measures are particularly useful, or generate theoretical explanations behind this variability. However, this moderation does not threaten our conclusions, since all indirect measures were tested across the same domains and outcomes.

In all, our results suggest that indirect measures should not be treated interchangeably, and our data align with the notion of substantive differences across measures in how each assesses a larger implicit construct. However, it will be essential for future work to better reveal and explain these differences; for instance, one possibility is that these measures vary in their capacity to tap into certain automatic processes, which would then reveal itself as variation in predictive validity. Supporting this explanation will require more focused follow-up work, either through the use of experimental manipulations that promote or weaken automatic responding across measures, or through advanced analyses that can offer more fine-grained inferences about what psychological processes are most active in determining performance within each measure.

### Conclusion

Researchers aware of various measures of implicit associations may feel as if they are interchangeable. However, our analyses revealed strong variation across measures for research conclusions concerning predictive and incremental predictive validity. The SC-IAT outperformed other indirect measures, likely due to its focus on associations toward stigmatized group members. Though many measures may tap into a larger implicit construct, those including associations toward non-stigmatized group members may introduce a diluting force that weakens predictive power toward important outcomes. Work in implicit social cognition will benefit from greater thought into not only the content of these influential measures but also toward selecting measures that are best aligned with our research questions.

## Appendix

**Appendix.** Category Labels and Item Stimuli by Topic Condition.

	Attitude category labels (for IAT, SC-IAT, SPF)		Attitude item stimuli (exemplars in IAT, SC-IAT, SPF; primes in EPT)	
Domain	Label 1	Label 2	Stimuli 1	Stimuli 2
Age	Old People	Young People	Images of old people (three males, three females)	Images of young people (three males, three females)
Arab	Arab Muslims	Other People	Words (*Hakim, Sharif, Yousef, Wahib, Akbar, Muhsin, Salim, Karim, Habib, Ashraf*)	Words (*Ernesto, Matthais, Maarten, Philippe, Guillaume, Benoit, Takuya, Kazuki, Chaiyo, Marcelo*)
Disability	Disabled Persons	Abled Persons	Images related to disabled persons (crutches, wheelchair, guide dog, blind person with cane)	Images related to abled persons (walking, running, walking on a road, skiing)
Gender-Career	Female	Male	Words (*Rebecca, Michelle, Emily, Julia, Anna*)	Words (*Ben, Paul, Daniel, John, Jeffrey*)
Gender-Science	Female	Male	Words (*Mother, Wife, Aunt, Woman, Girl, Female, Grandma, Daughter*)	Words (*Man, Son, Father, Boy, Uncle, Grandpa, Husband, Male*)
Race	Black People	White People	Images of Black people (three males, three females)	Images of White people (three males, three females)
Religion	Judaism	Other Religions	Images related to Judaism (menorah, star of David, Dreidel, Shabbat)	Images related to other religions (Buddha, totem, New Testament, Hindu statue)
Sexuality	Gay People	Straight People	Images (two men, two women) and words (*Gay, Homosexual, Gay People*)	Image (man and woman) and words (*Straight, Heterosexual, Straight People*)
Weapons	Black People	White People	Images of Black people (three males, three females)	Images of White people (three males, three females)
Weight	Fat People	Thin People	Images of fat people (four males, four females)	Images of thin people (four males, four females)

*Note.* IAT = Implicit Association Test; SC-IAT = Single-Category Implicit Association Test; EPT = Evaluative Priming Task; SPF = Sorting Paired Features.

## Notes

1. We replaced these 17 items to maximize the possible number of items that showed a reliable correlation with each indirect measure, since many would consider this a precondition for testing for incremental predictive validity. Given prior analyses finding positive correlations in performance across indirect measures within a topic (e.g., Bar-Anan & Nosek, 2014), replacing these items did not necessarily benefit one measure over any other.

2. This interpretation is supported by the finding that, for items where Evaluative Priming Task (EPT) and Sorting Paired Features Task (SPF) found evidence of incremental predictive validity, the correlation between structural equation modeling and OLS coefficients was high (EPT *r* = .90, SPF *r* = .97).

3. Only six items exclusively mentioned dominant group members, and 22 items did not directly mention either dominant or minority group members. For instance, one weight-related belief item asked participants to indicate the degree to which they agreed with the statement, “one’s weight is, to a great extent, controlled by fate.”
